# Microcrack monitoring and fracture evolution of coal and rock using integrated acoustic emission and digital image correlation techniques

**DOI:** 10.1038/s41598-024-58873-3

**Published:** 2024-04-05

**Authors:** Zhihui Zhao, Jinhu Yang, Yueming Kang, Yong Xiao

**Affiliations:** China Coal Technology Engineering Group Chongqing Research Institute, Chongqing, Chongqing, 400039 China

**Keywords:** Uniaxial compression, Mechanical characteristics, Failure mechanism, Crack evolution, And experimental analysis, Energy science and technology, Engineering

## Abstract

The mechanical properties of a coal–rock body were examined through uniaxial compression tests, and the rupture process of the coal–rock body was monitored in real time using a combined acoustic emission (AE) monitoring system and a digital image correlation (DIC) full-field strain measurement system. From a comparison of the mechanical properties of coal and sandstone, clear differences are apparent regarding the uniaxial compressive strength, deformation characteristics, and damage mode; the brittle failure characteristics of the coal samples are also more evident. The change in AE energy reflects the accumulation and release of elastic energy during the rupture process, and the evolution of AE localization points under different stress levels can effectively reflect rupture propagation. Further, the DIC full-field strain measurement method can quantitatively monitor the evolution of the displacement and strain fields at the marking point and surface simultaneously, thereby overcoming the limitations of traditional empirical and qualitative rupture processes. During monitoring, the AE focuses on the internal rupture of the specimen and the DIC focuses on the surface deformation. These complement each other and reflect the rupture process more comprehensively.

## Introduction

The mechanical properties and rupture laws of coal and rock have remained a hot topic of research in underground engineering, particularly in recent years^1–3^. Macroscopically, the rupture of a rock body includes the formation and penetration of cracks, and is often accompanied by a series of acoustic emission (AE) phenomena. Microcracking activity also inevitably influences the problem of energy, in which the generation and expansion of cracks involve the accumulation and release of strain energy. Therefore, using the combination of AE technology, digital image correlation (DIC), and other technological means of specimen rupture process monitoring^4,5^ the load-bearing capacities of coal–rock bodies can be characterized considering the mechanism of rupture and the law of evolution, which plays an important role in ensuring the safety of coal mines^6–8^.

Scholars have conducted numerous studies on the mechanical properties of coal–rock bodies as well as their deformation and rupture evolution laws, most of which were performed in a laboratory involving mechanical strength and deformation damage tests of coal–rock bodies^9–11^. Kong et al.^12^ conducted uniaxial compression tests on coal samples containing original fissures, and collected AE signals during the rupture process and crack expansion. Li et al.^13^ used an established acoustic-electrical-thermal multiparameter test system to observe the AE characteristics, surface potential, and infrared thermal radiation during the damage process of a coal–rock mass. Xiao et al.^14^ employed an AE monitoring system to collect signals corresponding to AE count, energy, and spectrum during the destruction of coal samples and noted that different loading stages presented different characteristics. Wang et al.^15^ analyzed the AE characteristics of rock-like materials under nonuniformly distributed loads; as a result, the AE characteristics and mechanical behaviors exhibited obvious localized partitions. Xu et al.^16^ analyzed the energy rate, cumulative energy distribution, and spatiotemporal evolution of AE under different stress levels. Cao et al.^17^ found a clear correspondence between the AE counting curves and the rock stress–strain curves, and that the AE energy increases significantly with an increase in the loading rate regarding increasing peak strength and elastic modulus. Sun et al.^18^ investigated the relationship between the stress and AE of a coal–rock body under multilevel dynamic loading. Liu et al.^19^ investigated the AE signals of coal rupture under uniaxial compression, and analyzed the spectral and energetic characteristics of the AE waveforms under different stress levels. Considering DIC methods for monitoring the fracture of coal–rock bodies, Yang et al.^20^ examined the crack extension process of unsaturated mudstone at low strain rates via DIC, while Munoz and Taheri^21^ obtained the full-field strain evolution during uniaxial compression of sandstone samples with different aspect ratios using the 3D-DIC technique. Pan et al.^22^ carried out uniaxial compression tests on pre-cracked rocks using a combined AE testing system and 3D-DIC techniques to analyze the peak strength before reaching the strain field evolution characteristics. Lotidis et al.^23^ conducted uniaxial compression tests on rock samples containing circular holes, and adopted AE and full-field digital image techniques to monitor the rupture and spalling processes. Liu et al.^24^ analyzed the full-field strain evolution of a single-fractured marble in uniaxial compression using DIC methods, and determined that the respective full-field strain evolution is not a significant factor in the strain evolution. This indicated that a strain localization feature appeared near the cracks.

From the above literature, certain differences are apparent in the mechanical properties of a coal–rock body; meanwhile, to monitor the rupture process of a coal–rock body, more mature means of testing, including acoustic, optical, and electromagnetic. The joint use of these techniques can reflect the rupture process of a coal–rock body more comprehensively, allowing better study of the damage mechanism of coal–rock and the law of crack. In this work, with the aim of revealing the failure mechanism of coal and rock mass, a uniaxial compression test was conducted to investigate the mechanical properties, and deformation and rupture evolution characteristics of a coal–rock body. The test was performed to determine the mechanical properties of standard coal–rock body specimens, while the uniaxial compressive strength, modulus of elasticity, Poisson’s ratio, and other basic mechanical parameters can be obtained according to the characteristics of the stress–strain curves. Further, an AE monitoring system and Digital Image Correlation (DIC) were used in combination for real-time monitoring of the damage process of the coal–rock body, analyzing the internal AE response characteristics of the specimen and the digital scattering full-field strain law on the surface during the rupture process, and examining the load-bearing mechanism and rupture mechanism of the coal–rock body in comparison with the experimental phenomena.

## Experimental materials and equipment

### Sample preparation

**Figure 1:**
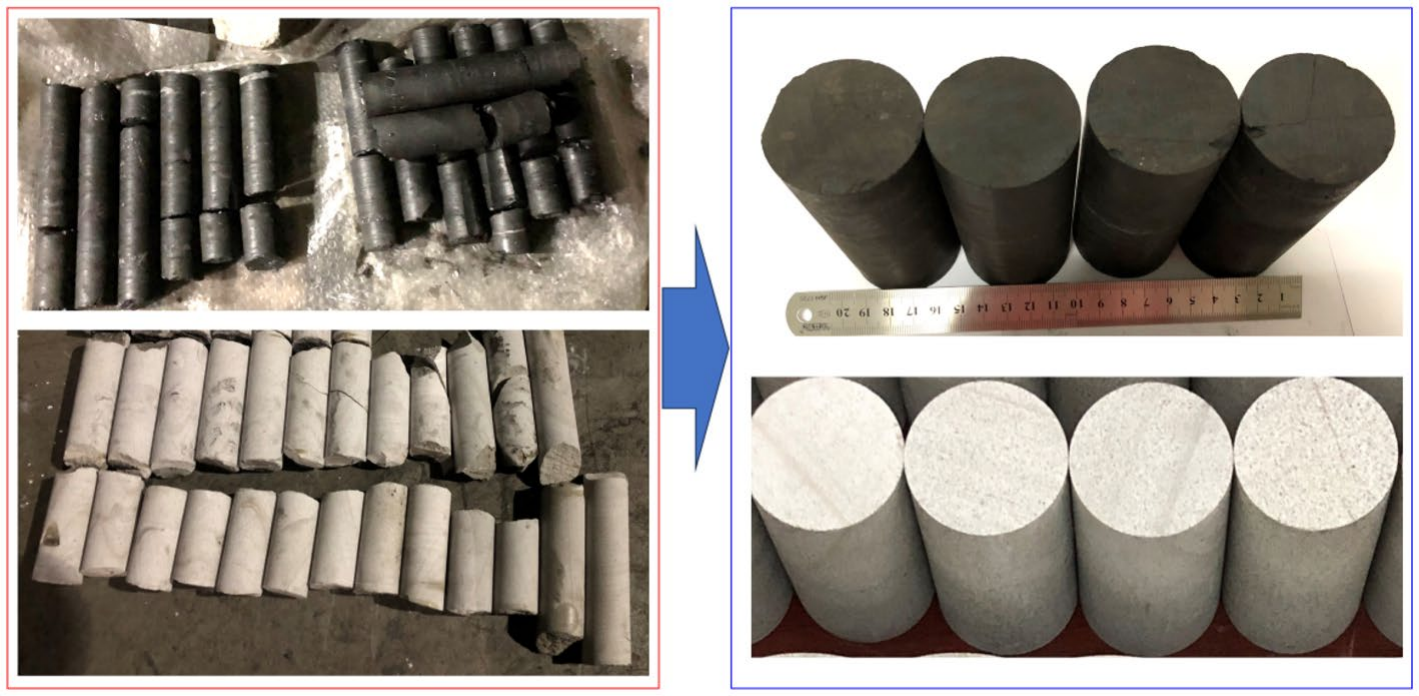
Test specimen.

The specimens used in the uniaxial compression test included two types of coal and rock, divided into two groups according to the lithology, each of which comprised four specimens, conforming to the regulations in the relevant test requirements^25^. Meanwhile, to facilitate the test records and data processing, the specimens were labeled M or R according to their type, M for the coal specimens and R for the rock specimens, as shown in Fig. 1 below. It is worth mentioning that the specimens used in the test were taken from the 8119 working face and roof sandstone of No. 3–5 Coal seam in Tashan Coal Mine, Datong City, Shanxi Province, with a buried depth of 436 m. All the coal and rock specimens were processed into standard cylinders with the diameter of 50 mm and height of 100 mm, resulting in the height-to-diameter ratio of 2:1, according to the standards recommended by the International Society for Rock Mechanics (ISRM)^26^. In addition, the unevenness of the upper and lower end surfaces of each specimen was less than 0.01 mm and the deviation of the end surfaces from the perpendicularity of the axes did not exceed 0.25°. Next, the fabrication process of each specimen type is introduced and discussed below.

In the manufacturing of specimens, to minimize the test error, all the test samples were taken from the same level of the large coal–rock body: a larger volume of good integrity was selected with no obvious cracks on the surface of the block. During transportation, a plastic film was used to minimize the damage to the package. Coal–rock specimens were then formed in the laboratory via core drilling, cutting, and polishing of large blocks of coal and sandstone taken from underground, using a drilling rig to drill in the same direction to ensure the consistency of the joints; a vertical drilling rig of type DJ2230 was used to drill the large blocks of coal–rock, with a customized diamond thin-walled drill bit; a special fixture was used to fix the whole specimen when coring; a tap-water cycle was used to cool the drill bit during the drilling process, and plastic film was used to wrap it to minimize damage. For drilling the specimen, tap water circulation was incorporated to cool down the drill bit, and then the specimen was cut with a cutting machine to the length of the excess part. The end was smoothed using a double-end grinding machine. Finally, the specimen was screened using Vernier calipers, and the specimen that met the standard was finally used for the uniaxial compression test.

### Experimental apparatus and procedures

**Figure 2:**
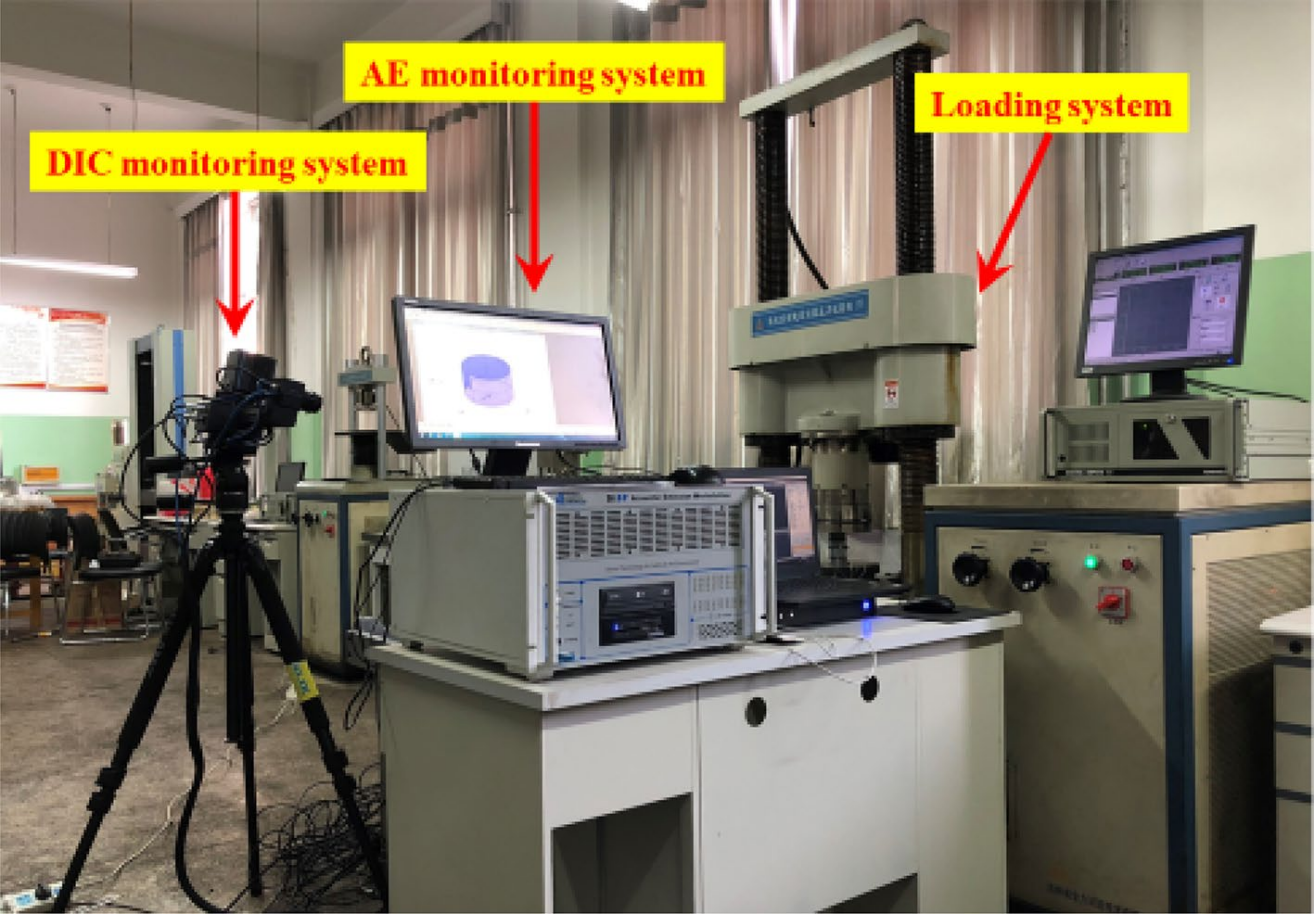
Test equipment.

The equipment used for the uniaxial compression test is depicted in Fig. 2 below, and mainly comprised a WAW-1000 microcomputer-controlled electro hydraulic servo pressure loading system, PCI-2 AE monitoring system, and XTDIC three-dimensional full field strain measurement system, which, according to their own functional characteristics, play different roles in the experimental process. The servo press was mainly used to provide the axial load, and the AE monitoring system was used to monitor the AE signals during the damage process. To intuitively respond to the rupture evolution on the surface of the specimen, a three-dimensional full-field strain measurement system was used to trace the rupture extension process on the surface in real time, and to obtain the full-field strain and rupture evolution of the specimen surface during uniaxial compression. It is worth stating that the testing equipment should be turned on synchronously during the test and analyzed by time in later data processing.

1. Microcomputer-controlled electrohydraulic servo pressure loading system
  The testing machine can realize fully digital closed-loop servo control, its maximum bearing capacity is 1000 kN, the displacement measurement resolution can reach 0.002 mm, and its loading frame has sufficient strength and stiffness to complete the coal–rock compression test, which includes displacement control and force control. The test was placed in the center of the pressure head, and the displacement loading mode of 0.002 mm/s was carried out until the specimen was destroyed.
2. Acoustic emission (AE) monitoring system
  A 16-channel AE monitoring system manufactured by Physical Acoustics (PAC) Inc. was adopted to observe the specimen. The probe, amplifier, and collector were connected through cables, and the threshold was set to 45 dB at the sampling rate of 1 MSPS. To locate the internal rupture point of the specimen, we installed eight nano30 probes (bandwidth of 125–750 kHz, resonance frequency of 140 kHz) around the specimen, and white Vaseline was applied to the probe surface and the contact surface of the specimen to enhance the coupling effect, which were clamped with an elastic band to achieve good fit with the specimen.
3. Full-field strain measurement system
  The digital scattering DIC measurement system produced by Xi'an Xintuo 3D Optical Measurement Technology Co., Ltd. was adopted, and consists of a high-speed camera, image acquisition card, and calculation and analysis software; the 12 MP dual camera for which is from Basler Company, Germany, the frame rate of acquisition was set to 75 fps, and the time between sampling interval was set to 1000 ms. First, black and white digital scattering spots were sprayed on the surface of the specimen for identification and tracking, and then the specimen was placed in the center of the indenter for fixation after the spots dried. The initial coordinates were calibrated using the calibration plate before the test, the camera captured images during the test, and finally, the three-dimensional coordinates of the specimen, the displacement field, and the strain field were analyzed using the image processing and calculation software.

## Experimental results and analysis

### Stress–strain curves

**Figure 3:**
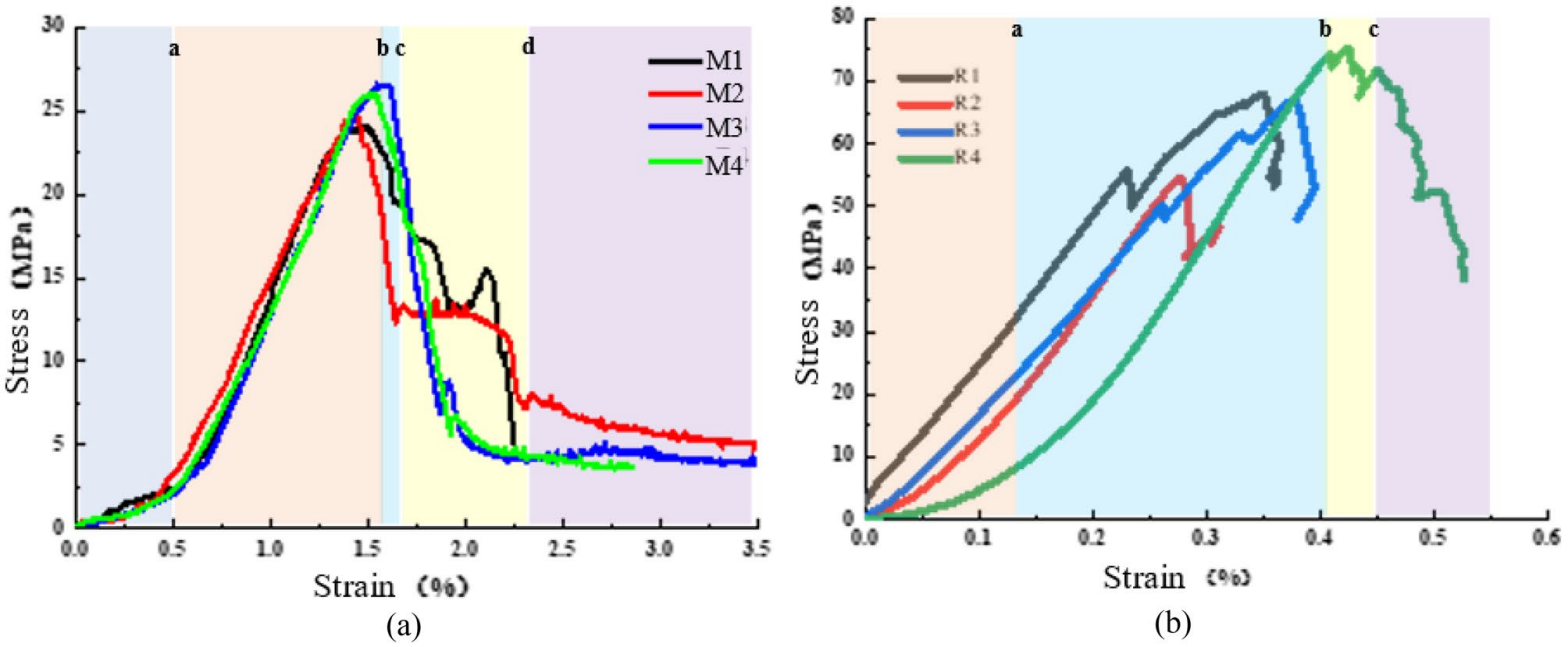
Full stress–strain curves of specimens. (**a**) coal (**b**) rock.

The stress–strain curves of the coal–rock specimens under uniaxial compression are displayed in Fig. 3; the deformation and destruction processes present apparent stages, which can be divided into four: nonlinear compaction, linear-elastic deformation, pre-peak plasticity, and post-peak strain softening. The initial stage is the nonlinear compaction stage, ranging from the beginning of the load to point a. Owing to the high development of the internal pore and microcrack structure of the coal, which leads to the obvious nonlinearity of this stage, the duration is long; at the end of this stage, the original defective structure has been closed, and the second stage is the linear elastic deformation stage (from a to b), which shows a linear growth of the stress along with an increase in the strain. Meanwhile, elastic energy gradually accumulates in this stage, and this stage accounts for a larger proportion of the entire curve. The third stage is the peak before the plastic deformation stage in the curve between point b and the peak stress point c, owing to the fluctuation of the curve at this stage, and the duration of the time is shorter so it is difficult to distinguish between the individual specimens. The cracks begin to propagate gradually at this stage. When the stress reaches the peak point c into the strain-softening stage, at this stage, with the increase in strain, the stress suddenly decreases. The strain increases, the stress suddenly decreases, and the elastic energy accumulated in the early stage is suddenly released, showing obvious brittle failure behavior. There is almost no residual strength of the damaged specimen, while the sudden release of elastic energy is manifested as obvious brittle damage.

The full stress–strain curves of sandstone specimens under uniaxial compression are shown in Fig. 3, and the deformation damage process can be divided into five stages according to the change of the slope of the curve, in which the compaction stage, linear elasticity stage, and plastic deformation occur before the peak, and the interval from the beginning of the loading to the a-point is the compaction stage. The stress–strain curves within this stage present an up-concave shape, and the increment of the strain decreases with the increase in stress. This is mainly due to the closure of microcracks and pores inside the specimen. The second stage is the linear elasticity stage from point a to point b. In this stage, the strain increases linearly with the increase in stress, exhibiting obvious linear elastic behavior, in which the elastic modulus E can be obtained from the slope of the straight line at 50% of the peak stress. Most of the total energy accumulated in the specimen is converted into the elastic energy of the rock before reaching the peak point, and the deformation will be reduced with the elastic energy of the specimen if unloading of stress is carried out at this stage. deformation disappears with the gradual release of elastic energy and resumes deformation. The third stage is the plastic deformation stage, from the end of the linear stage b to the peak stress point c. At this time, the stress–strain curve presents an obvious upward convex shape, with gradual increase in stress–strain in the phenomenon, indicating that the microcracks began to expand steadily to large cracks, and the volume of the specimen in the expansion stage of the deformation of the internal structure of the change is irreversible; at the end of the plastic deformation, the strength reaches its peak value. At the end of plastic deformation, the strength reached its peak value and then entered the post-peak deformation stage.

### Uniaxial compressive strength

According to the above stress–strain curve to calculate the mechanical parameters of coal–rock, specified in Table 1, where *E* represents the modulus of elasticity obtained by the slope of the stress–strain curve in the elastic phase, and *μ* is Poisson's ratio, i.e., the ratio of axial strain to radial strain; *σ*_p_ represents the peak strength, i.e., the ratio of the maximum load carrying capacity to the cross-sectional area, and *σ*_r_ is the residual strength to represent the load carrying capacity of the specimen after the destruction of the specimen, which is usually used as a combination of the two indicators of the peak and residual strength to reflect the specimen’s strength characteristics and load carrying capacity.

**Table 1:** Experimental results of mechanical parameters.

Type	Number	Elastic *E* (GPa)	Poisson's ratio *μ*	Peak stress *σ*_p_ (MPa)	Peak strain *ε*_p_	Residual stress *σ*_r_ (MPa)	Residual strain *ε*_r_
Coal	M1	10.85	0.31	9.80	1.16	0.16	1.34
	M2	10.15	0.26	8.61	1.41	0.36	1.56
	M3	9.75	0.27	6.43	1.10	0.58	1.81
	M4	10.25	0.28	8.28	1.22	0.367	1.57
Rock	R1	27.84	0.24	24.13	1.50	4.23	2.53
	R2	29.52	0.19	24.72	1.86	5.20	3.93
	R3	27.18	0.23	27.41	1.30	5.04	3.37
	R4	28.18	0.22	25.42	1.55	4.82	3.28

By analyzing the data in Table 1 above, the average modulus of elasticity for coal and sandstone is 10.43 and 26.72 GPa, respectively, and the strength of coal is lower than that of sandstone, with average peak strengths of 8.97 and 25.30 MPa, respectively, while the average residual strengths of the coal and the rock specimens are 0.37 and 4.82 MPa, respectively. The value for the sandstone is approximately 10 times higher than that of the coal, indicating that the sandstone after destruction may be different than coal, and sandstone has a larger residual bearing capacity.

### Failure characteristics

**Figure 4:**
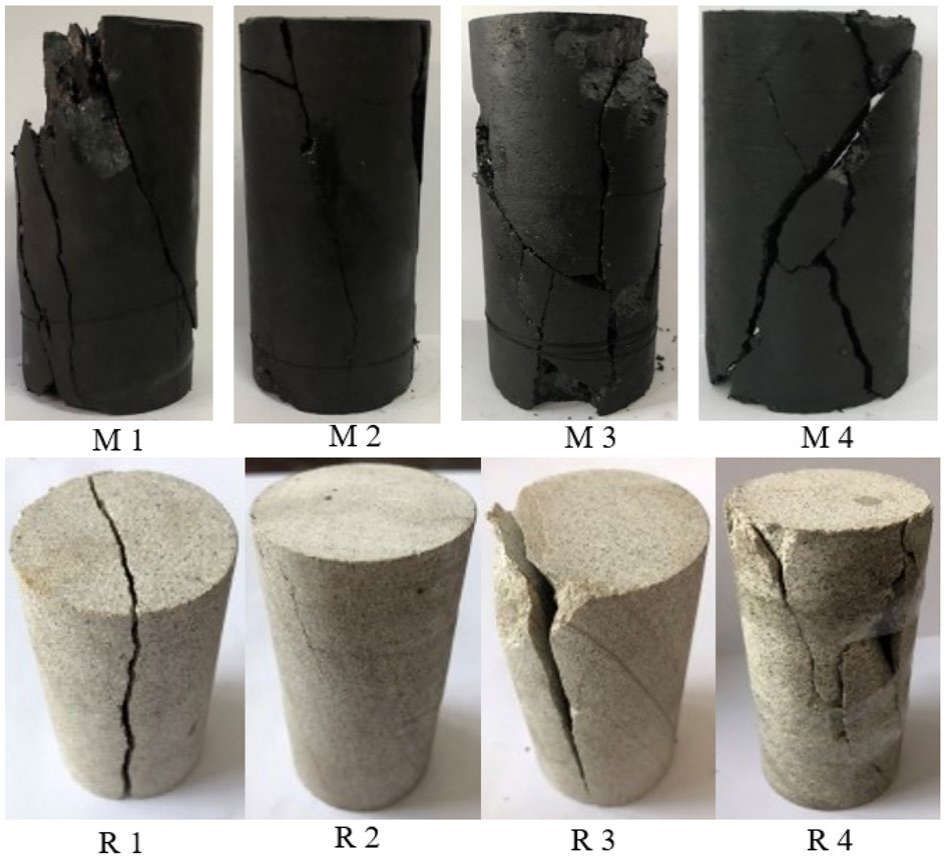
Failure modes of samples.

The damage characteristics of the coal–rock specimens are shown in Fig. 4. The scattered pieces of coal–rock after destruction were combined and recovered, and the larger cracks were sketched to observe the damage pattern. The main failure mode can be inferred through the analysis of the instant of destruction and rupture characteristics of the coal–rock body.

In the test process, the sandstone specimen did not make an obvious sound when destroyed, the degree of fragment splashing after destruction was much smaller than that of the coal specimen, and there was no sudden failure phenomenon in the late stage of the test. Further, the integrity of the specimen after destruction was better, and only a small piece of specimen fell off while the main cracks existed. This also shows that the specimen maintains a certain bearing capacity after damage, which is consistent with the previous full stress–strain curve peak after a certain residual strength.

As can be seen from the damage characteristics of the spliced coal and the sketch diagram below, several obvious inclined fracture surfaces appeared in the damaged coal samples, indicating that the damage form is mainly shear damage. Under the action of compressive stress, the specimen slipped along the shear surface, of which the M1 specimen showed two obvious parallel fracture surfaces, presenting obvious double-shear damage characteristics. The M2 specimen was distributed on a single fracture surface across the upper and lower ends, presenting obvious single-shear damage characteristics. In contrast, the M3 specimen was relatively broken, in addition to the central distribution of an obvious inclined fracture surface, and many secondary cracks distributed, presenting as an irregular “X”. The M2 specimen has a single fracture surface across the upper and lower end surfaces, showing obvious single shear damage characteristics; in contrast, M3 specimen is relatively broken, in addition to the distribution of an obvious tilted fracture surface in the middle of the distribution of the distribution of the many secondary cracks, showing an irregular "X"-type damage pattern; on the M4 specimen, there are two obvious tilted cleavage surfaces, one of which is a crack from the upper right corner of the specimen to the lower left corner of the expansion, and can be seen during the rupture process. Moreover, two obvious inclined fissures appeared on the M4 specimen surface, one of which extends from the upper right corner to the lower left corner of the specimen, and it can be seen that obvious deflection occurs during the rupture process, and the other fissure surface extends from the central nuclear area to the lower right corner, and obvious nucleation phenomenon occurs in the center. The damage pattern presents obvious "Y"-shaped damage characteristics, and it can also be seen that there are more fragments of the coal–rock after the damage than the sandstone, and it is different from the sandstone specimen. In addition, it can also be seen that there are more fragments after the damage than sandstone, and different from the sandstone specimen, there is a sudden damage phenomenon in the test, which coincides with the sudden drop after the peak of the full stress–strain curve in the previous section, indicating that the accumulated elastic energy is suddenly released when reaching the load-bearing limit. Meanwhile, the integrity of the specimen after the damage is relatively poor, and the shear slip surface can hardly withstand the loading, resulting in a very low residual strength.

Under the action of uniaxial compression, the microcracks inside the rock gradually nucleate, expand and extend, and eventually form macro cracks. Overall, it can be seen that the sandstone is mainly dominated by two modes of shear damage and longitudinal cleavage damage, with a larger size of the granularity after the rupture. Among them, the R1 and R3 specimens present obvious longitudinal through cracks, the form of destruction is mainly dominated by tensile damage, under the action of longitudinal compressive stress transversely appear tensile stress, microcracks along the longitudinal direction of the gradual expansion of the propagation, presenting obvious longitudinal cleavage damage characteristics; after the destruction of specimen R2, cracks are not obvious, cracks from the top upper-left corner of the tip to the lower right gradually expand, but cracks do not run through the entire specimen R4 specimen has obvious penetration of the tilted fracture surface, showing a clear "Y" type damage mode, and specimens R1 and R3 can be seen in comparison with the specimen that the fracture surface is more irregular, from the surface of the specimen on the right side of the flaking debris, in the process of destruction there may be a gradual destruction of the phenomenon. Progressive damage can occur during this process.

## Failure process and fracture evolution

### AE response characteristics during failure process

**Figure 5:**
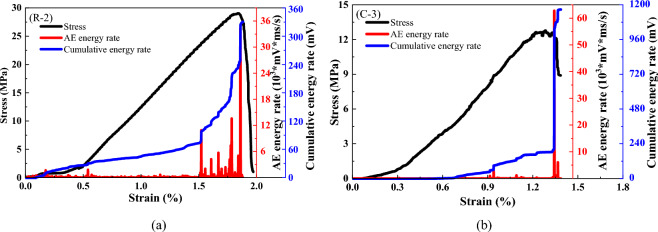
Energy evolution of the coal and rock failure processes. (**a**) coal (**b**) rock.

As the AE energy and cumulative energy can reflect the energy release during the damage process of the specimen, Fig. 5 depict the AE energy release with the change in the loading process for the coal and sandstone specimens. It is apparent that in the initial loading of the compression stage, the acoustic energy is very little, with the increase in load into the elastic phase, the AE energy release is relatively small mainly stored energy, while the AE cumulative energy linearly increased. When the peak specimen ruptures, accompanied by the release of energy, the energy surges after the peak stress drop, but the energy is significantly greater than the pre-peak stage, indicating that the peak specimen will still rupture and is accompanied by the release of energy when the gradual destruction of the energy phase is released.

The comparative analysis shows that the energy change trends of sandstone and coal are similar, both in the initial compaction and linear elasticity stages. The AE energy release is less and uniformly distributed before the peak stress, and the accumulated energy increases with the increase in stress. When the stress increases to near the peak stress, the AE energy of the coal and rock specimens reaches a maximum, and its AE characteristic law can be attributed to the following points:

1. In the unaffected stage, i.e., when the specimen is loaded at the initial stage, the specimen does not produce significant AE event counts because both the AE event counts and energy are low. A large number of primary cracks already existed inside the specimen in the original state, and after the initial loading, the microcracks were gradually compacted; however, because the internal changes were not very obvious, the number of AE events in this stage was relatively small, and the intensity was correspondingly low.
2. In the transition stage, with a gradual increase in stress, AE events began to appear and were characterized by a long duration, but the count and intensity were still relatively low. At this time, the specimen was subjected to closure of the fissure, which led to the slipping of the rough surface of the fissure, increasing the friction of the AE events, but had not yet reached the maximum degree.
3. In the active stage, with increasing stress, the AE events show a sharp increase in the phenomenon. The AE events under this stage are more frequent, and the parameters are shown at a higher level. The specimen in this state produced obvious macroscopic cracks, and simultaneously, the energy inside the specimen was suddenly released after a large amount of energy.
4. In the decay phase, when the specimen was destroyed, part of the block still had a certain bearing capacity; however, its strength was not very high, which ultimately led to a decline in energy and slowly disappeared.

Deformation and rupture will occur when the coal–rock body is subjected to force, and the stored strain energy will be released in the form of elastic waves during the rupture process, while the spatial localization of the AE events can intuitively reflect the location of the rupture within the coal–rock body and the process of change, and the aggregation and distribution of the rupture points within the specimen change with the generation, expansion, and penetration of the cracks, as shown in the figure below for the localization of the spatial and temporal evolution characteristics of the different loading stages of the coal and the sandstone, which shows that the AE characteristics of the coal and the rock differ obviously under the same test conditions and that the number of the AE events of the rock samples is obviously fewer than that of the coal samples.

**Figure 6:**
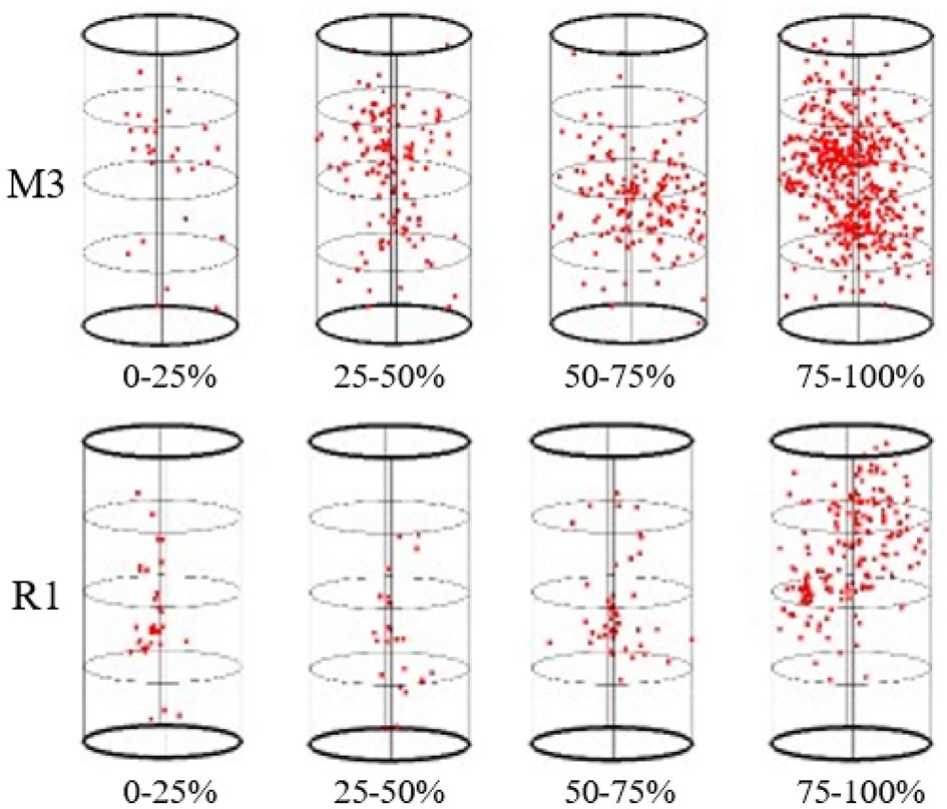
AE spatial–temporal evolution of the M3 and R1 specimens.

As Fig. 6 shows the spatial evolution of AE localization events in coal specimen M3, when the initial stress level is relatively low placed in the intervals 0–10%, 10–20%, 20–30%, and 30–40%, the AE localization points generated in the whole specimen space are few and randomly distributed, which may lie in the inhomogeneity of defects in the coal, and with the increase of the stress level, the rupture points in the spatial distribution evolution is also increasing, in which the number of AE events increases significantly in the stress intervals of 40–50%, 50–60%, 60–70%, and 70–80%, and the distribution of new localization points is concentrated in the middle of the specimen, and the AE localization points almost cover the whole specimen when the stress intervals of 80–90% and 90–100% are reached, and in the stress interval of 90%–100%, the number of AE events reaches the maximum value, generally, before the stress peak. The distribution of AE localization points shows random characteristics, but unlike the pre-peak, the post-peak AE localization points arise near the shear fracture, such as in the stress intervals of 80–30% and 30–20%, which indicates that sliding motion along the shear surface may have occurred in the post-peak stage, and the damage of the coal is dominated by the shear failure, accompanied by many secondary microcracks, which is consistent with the morphology of the M3 specimen after final damage in the previous section.

The spatial evolution characteristics of AE localization points of sandstone R1 specimens at different loading stages are shown in Fig. 6, which show that the number of AE localization points increases gradually with the increase of loading before reaching the peak value and reaches the maximum value when the peak load is reached, where the initial loading incremental stages within the ranges of 0–10%, 10–20%, and 20–30% produce a relatively small number of AE locating points, which were mainly dispersed around the central axis in the middle of the specimen, and the number of AE locating points increased slowly and were randomly distributed on both sides of the central axis as the stress intervals were increased to 30–40%, 40–50%, and 50–60%. When the stress intervals reached 60–70%, 70–80%, and 80–90%, the AE events gradually increased and nucleation zones of AE location points were formed in the middle and lower parts of the specimens, and when approaching the peak stress intervals of 90–100% and 100–90% on both sides of the peak stress intervals, the AE events increased rapidly and reached the maximum value, which were distributed longitudinally around the central axis, and the rupture points were mostly distributed on the central axis. The previous section presents a longitudinal splitting damage pattern consistent with the previous section. Randomly distributed AE rupture points still exist in the descent and residue of the post-peak phase.

A comparative analysis shows that the spatial evolution of the AE localization points during stress loading can accurately reflect the damage characteristics of rocks and coal. In addition, the AE rupture characteristics of coal and rock are clearly different, and the number of AE rupture points in rocks is much lower than that of coal samples under the same experimental conditions, which may be related to the different damage modes and material properties.

### Digital speckle full field strain and dynamic rupture

**Figure 7:**
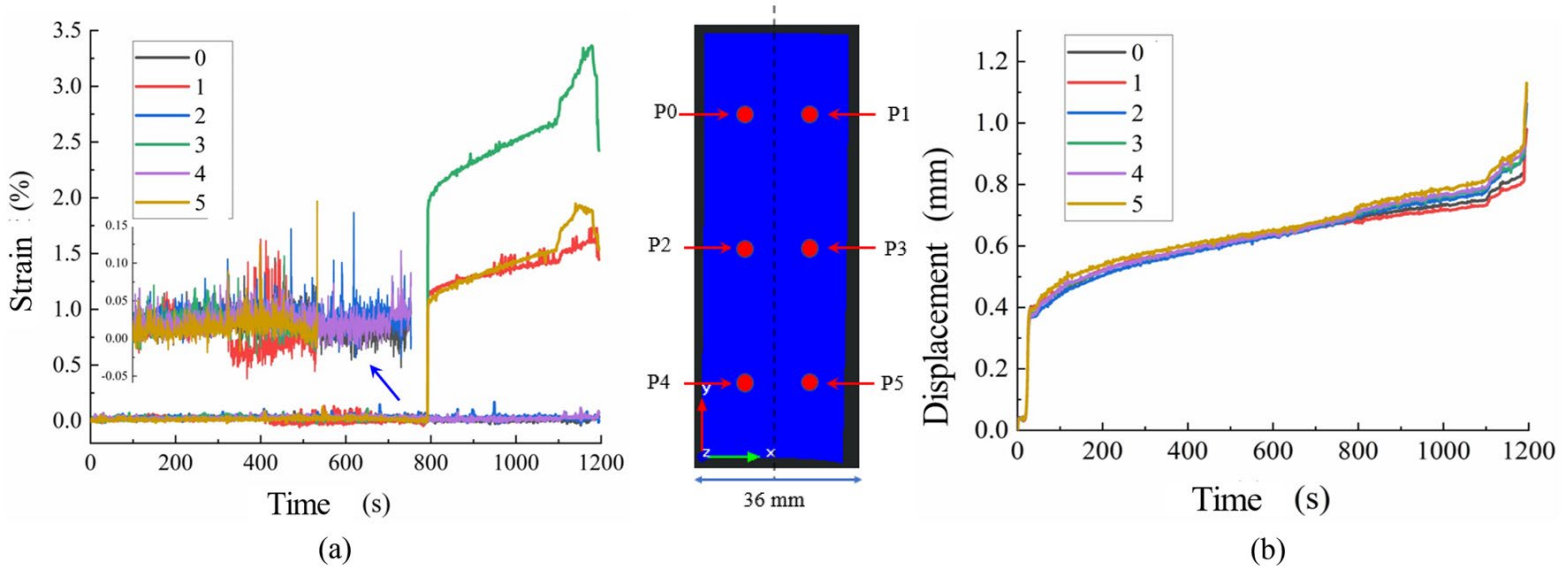
Deformation curves of marked points on specimen surface. (**a**) Strain (**b**) Displacement.

The displacement and strain curves of the marked points on the surface of the specimen are shown in Fig. 7; the displacement curves of the six marked points during the rupture process of the specimen exhibited the same trend of change, presenting a nonlinear growth trend, and their morphology was similar to that of a typical creep curve of a coal–rock body. According to the slope of the curve, the displacement and deformation can be divided into three stages: the initial stage, isochronous deformation stage, and accelerated deformation stage. The interval of the initial deformation stage is in the range of 0–200 s, and the deformation rate decreases with an increase in time, which may be caused by the existence of more pore cracks inside the coal–rock body and greater compressibility of the pore cracks in the initial compression stage. The interval is 200–1000 s, the displacement in this stage shows linear growth with time, and the deformation rate is basically stable, the initial pore cracks in the coal–rock body have been basically compacted in this interval, but with the gradual increase of the stress the pore cracks are expanding; the third accelerated deformation stage is from 1000 s to the end of the test, and the deformation rate gradually increases in this interval with time, and reaches the destruction of the coal–rock body. The third stage of the accelerated deformation stage is from 1000 s to the end of the test, in which the deformation rate increases with time and shows a steep increase when reaching the damage. With the increase in stress when reaching the peak load, the cracks expand rapidly and form fracture surfaces, which leads to the acceleration of the deformation.

Through the strain curve of the marked points on the surface of the specimen Fig. 7, it can be found that there is a certain difference between the strains on both sides of the specimen in the process of deformation and destruction, and the strain on the right side is larger than that on the left side. Moreover, from the distribution of the marked points above, it can be seen that monitoring points 1, 3, and 5 are distributed on the right side of the specimen, while monitoring points 2, 4, and 6 are distributed on the left side of the specimen, and the differences can be seen according to the strain–time curves of the destructive process, in which the strain at the six points in the range of 0–800 s exhibits basically the same trend, as can be seen from the magnified figure, they are basically in the vicinity of 0.03 up and down fluctuations. As the loading of the fluctuation amplitude increases, and the maximum fluctuation value reaches 0.15; when it reaches approximately 800 s, the strain of monitoring points 1, 3, and 5 appear to steeply increase, the amplitude for which of monitoring points 1 and 5 is basically the same, with the strain value of 0.15. The magnitude of the steep increase in strain at monitoring points 1 and 5 was essentially the same, and the strain value increased to approximately 1.0, whereas the magnitude of the steep increase in strain at monitoring point 3 was larger than those at monitoring points 1 and 5, and the strain value increased to approximately 2.0. In addition, comparing the displacement curve, it can be seen that the displacement rate also increases at this time, but the specimen is not damaged at this time. Thus, the strain of monitoring points 1, 3, and 5 shows a linear growth trend in the subsequent 800–1100 s interval, and there is a significant increase in the strain rate of monitoring points 1, 3, and 5 in the interval from 1100 s to the end of the test and a reduction in the specimen damage, whereas the strain of monitoring points 1, 3, and 5 on the left side of the specimen is the same. In contrast, monitoring points 2, 4, and 6 on the left side of the specimen showed fluctuating changes in strain from the beginning to the end of the test, and the amplitude of fluctuation increased with an increase in stress. However, there was no steep increase in the trend of change, which also indicates that the strain field on both sides of the monitoring surface exhibited nonuniform deformation characteristics.

**Figure 8:**
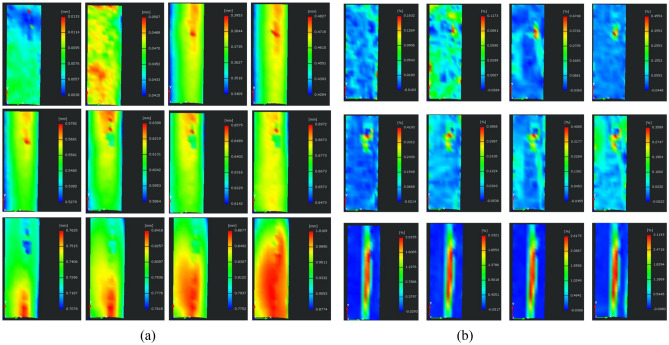
Evolution of deformation field on specimen surface. (**a**) Displacement field (**b**) Strain field.

As shown in Fig. 8, for the monitoring of the displacement field image sequence, the sequence can be analyzed through the dynamic process of specimen rupture. It can be seen that in the loading of the initial specimen surface displacement field in the entire face of the random distribution, owing to the loading of the initial specimen internal distribution of the uneven distribution of the air cracks. Then, in the specimen above the central region, displacement increases, and there is a clear displacement of the region of the high displacement. Then, the displacement distribution is gradually reduced from top to bottom. At this time, the displacement distribution in the surface is gradually decreasing from top to bottom, and the displacement on the right side is larger than that on the left side in the transverse direction; however, with the continuous increase in stress, the maximum displacement zone extends downward, showing a large middle and two sides of the small morphology, and the maximum displacement distribution presents a parabolic shape, while the displacement difference between the two sides is gradually leveling off; with the transfer of the high-displacement zone downward in the middle of the surface to form a strip-like high displacement zone transition zone. As the high-displacement zone shifted downward, a strip-like transition zone was formed in the middle of the surface; thereafter, a high displacement began to appear in the bottom center of the specimen and continued to extend upward until the specimen was damaged. In summary, it can be seen that the strain in the center is always greater than that in the two sides, which coincides with the damage pattern of cleavage, and in the process of rupture, the high displacement zone appears to be transferred, first from the top to the bottom, and then from the bottom upward extension until destruction. It can be seen from the cloud diagram of the maximum principal strain that the maximum principal strain on the observation surface distributes randomly at the initial stage of loading. With the increase of load, a strain concentration area begins to appear in the local area on the upper right of the observation surface, and the maximum principal strain expands around the nuclear region, but the local strain concentration area does not affect the fracture of the specimen. When the specimen is on the verge of failure, the maximum principal strain is mainly concentrated in the middle of the specimen, and presents a longitudinal banded distribution, gradually decreasing from the edge of the strain concentration area to the top and bottom ends, which is consistent with the splitting failure mode and displacement evolution cloud map presented by the specimen.

Comprehensive displacement field and the evolution of the strain field can be found, the development of cracks within the rock and rupture is the result of the accumulation of the previous period, the deformation of the specimen appears in the pre-loading period of the region is relatively random, mainly due to the specimen internal pore cleavage caused by the closure of the loading process in the early stage of the distribution of uneven distribution of most of the random distribution; then with the increase in the load began to form a concentration of localized areas, but due to the load at this time is relatively low the Concentrated area can only be called the potential rupture location, when the load is increased to near the peak, the high displacement zone began to expand steadily in one direction, and accompanied by the emergence of the local strain zone in the form of strips, at this time it can be determined that the region is the path of the specimen will be ruptured.

## Results and discussion

In this section, we discuss the difference of coal and sandstone from the perspective of comparative analysis based on the results obtained in "Experimental results and analysis" and "Failure process and fracture evolution". By comparing the mechanical properties of coal and sandstone, there were obvious differences between coal and sandstone in terms of uniaxial compressive strength, deformation characteristics and damage mode. In comparison, the brittle failure characteristics of coal sample were more obvious. By comparing the mechanical properties of coal and sandstone, it can be seen that there are obvious differences between coal and sandstone in uniaxial compressive strength, deformation characteristics and failure modes, among which the strength of sandstone is significantly higher than that of coal sample, and the former has a higher residual strength; There are obvious differences between the compaction stage and the plastic deformation stage of their stress–strain curves. The compaction stage of coal sample is larger than that of rock sample and presents obvious nonlinearity, while the plastic deformation stage near the peak of rock sample is larger than that of coal sample. In addition, the failure form of coal sample is more complex, and the fragmentation degree is obviously smaller than that of rock sample. Sandstone is mainly dominated by longitudinal splitting failure and shear failure, while coal sample is mainly dominated by shear slip failure, and the crack morphology after rupture mainly includes single shear plane, double shear plane and "X" type failure. Therefore, the brittle failure characteristics of coal samples are more significant in comparison. In addition, compared with the coal samples, it is clear that the stress–strain curves of sandstone significantly differ from those of coal, the proportion of deformation near the peak is significantly larger than that of the coal samples, and there are obvious post-peak deformation behaviors and residual stresses in sandstone, which are related to the differences between coal and rock in composition and pore and fracture structures.

The change in acoustic emission energy reflects the accumulation and release of elastic energy during the rupture process, and the evolution of acoustic emission localization points under different stress levels can effectively reflect the rupture propagation. Using DIC full-field strain measurement method can quantitatively monitor the evolution of the displacement and strain fields at the marking point and surface at the same time, which breaks through the limitations of traditional empirical and qualitative-based rupture process. In the monitoring process, the AE pays attention to the internal rupture of the specimen and the DIC focuses on the surface deformation. They complement each other can more comprehensively to reflect the rupture process.

## Conclusions

In this study, the mechanical properties of a coal–rock body were examined through uniaxial compression tests, while the rupture process of the coal–rock body was monitored in real time using a joint AE monitoring system and digital scattering full-field strain measurement and analysis system. The load-bearing and rupture mechanisms of the coal–rock body were analyzed according to the test results. The main conclusions of this study are provided below.

1. From a comparison of the mechanical properties of coal and sandstone, clear differences can be seen between coal and sandstone regarding the uniaxial compressive strength, deformation characteristics, and damage modes, in which the strength of sandstone is obviously higher than that of coal samples, and the former has a higher residual strength. There is a clear difference between the compression stage and plastic deformation stage of their stress–strain curves, with the compression stage of coal samples accounting for a larger proportion than that of rock samples and presenting obvious nonlinearities, while the proportion of rock samples in the plastic deformation stage near the peak is significantly larger than that of coal samples. There are obvious differences between the compression and density stage and plastic deformation stage of their stress–strain curves: the compression-density stage of coal samples is larger than that of rock samples and exhibits obvious nonlinearity, while the plastic deformation stage of rock samples near the peak accounts for a larger proportion than that of the coal samples. The crack patterns after rupture mainly include single shear surface, double shear surface and "X" type damage. Therefore, the brittle damage characteristics of the coal samples were more significant.
2. By analyzing the spatial evolution characteristics of the AE energy release and rupture point during the rupture process, it can be seen that the AE energy release during the damage process of the specimen is closely related to the process of germination, development, and penetration of the internal cracks: the AE energy starts to increase significantly at the end of the elastic phase, and the value of the AE energy increases to the maximum value at the moment of rupture of the specimen, accompanied by a sudden increase in the acoustic energy rupture localization point. The AE energy changes reflect the accumulation and release of the elastic properties during the rupture process, and the evolution of AE localization points under different stress levels can effectively reflect the rupture region and its direction of expansion.
3. The DIC full-field strain measurement method was adopted to quantitatively monitor the displacement field and strain field evolution process of the marking point and rupture surface, which overcomes the limitations of the traditional empirical and qualitative-based rupture processes, in which the deformation law of the marking point is similar to that of the creep curve and can be classified into initial deformation, isochronous deformation, and accelerated deformation in three phases. In addition, the displacement field of the monitoring surface reflects the process of fracture expansion from the bottom to the top, and the full-field strain field reflects the tensile strain generated in the middle of the specimen. The change and transfer of the strain field is the root cause of the emergence and expansion of cracks in the coal–rock body, and the localized strain field in the early loading stage will produce local cracks, which will have a certain impact on the rupture of the whole specimen. However, the change in the strain field in the late loading stage is more capable of reflecting the direction of crack expansion. The direction of the crack extension was also consistent with the damage mode of the final longitudinal splitting of the specimen.
4. According to the AE response and DIC full-field strain evolution characteristics of the rupture process of the coal–rock body under uniaxial compression, the AE characteristics mainly reveal the development process of the internal cracks of the coal–rock body and the law of energy evolution; whereas, the DIC full-field strain system mainly reveals the deformation law of the specimen’s surface displacement field and the strain field; the former monitors the internal rupture of the specimen and the latter monitors the deformation of the surface, which are complementary to each other and can reflect the rupture process of the coal–rock body in a more comprehensive and objective manner.

## Data Availability

The datasets used and analyzed during the current study are available from the corresponding author on reasonable request.
